# Hydrodynamic characteristics of knotted and knotless purse seine netting panels as determined in a flume tank

**DOI:** 10.1371/journal.pone.0192206

**Published:** 2018-02-08

**Authors:** Hao Tang, Liuxiong Xu, Fuxiang Hu

**Affiliations:** 1 College of Marine Sciences, Shanghai Ocean University, Shanghai, P. R., China; 2 School of Ocean and Earth Science, Tongji University, Shanghai, China; 3 National Engineering Research Center for Oceanic Fisheries, Shanghai, P. R., China; 4 Shanghai Education Commission “Summit and Highland” Discipline Construction for Fisheries Sciences, Shanghai, P. R., China; 5 Faculty of Marine Science, Tokyo University of Marine Science and Technology, Minato, Tokyo, Japan; Hampden Sydney College, UNITED STATES

## Abstract

Nylon (PA) netting is widely used in purse seines and other fishing gears due to its high strength and good sinking performance. However, hydrodynamic properties of nylon netting of different characteristics are poorly understood. This study investigated hydrodynamic characteristics of nylon netting of different knot types and solidity ratios under different attack angles and flow velocities. It was found that the hydrodynamic coefficient of netting panels was related to Reynolds number, solidity ratio, attack angle, knot type and twine construction. The solidity ratio was found to positively correlate with drag coefficient when the netting was normal to the flow (*C*_*D*90_), but not the case when the netting was parallel to the flow (*C*_*D*0_). For netting panels inclined to the flow, the inclined drag coefficient had a negative relationship with the solidity ratio for attack angles between 0° and 50°, but a positive relationship for attack angles between 50° and 90°. The lift coefficient increased with the attack angle, reaching the culminating point at an attack angle of 50°, before subsequent decline. We found that the drag generated by knot accounted for 15–25% of total drag, and the knotted netting with higher solidity ratio exhibited a greater *C*_*D*0_, but it was not the case for the knotless netting. Compared to knotless polyethylene (PE) netting, the drag coefficients of knotless PA netting were dominant at higher Reynolds number (Re>2200).

## Introduction

Meshes of traditional netting panels are composed of bars and knots. More recently, netting panels can be weaved without knots, and are called knotless netting. In knotted netting, single English knot and double English knot are commonly used knotted types for fishing nets, while in knotless netting, twisted Cross and Ultra Cross (named by Nichimo Co., Ltd, Shimonoseki, Japan) are the basic knotless type according to the way the mesh is wove. One parameter of a netting panel is the solidity ratio, which is defined as the ratio of projected area of bars and knots to the outline area of a netting panel. Different knot types influence the solidity ratio of netting, which may result in differential hydrodynamic forces. Usually the knotted netting has greater drag force than knotless netting of the same twine diameter and materials, and knotted netting panels with different knot types have different hydrodynamic characteristics [1].

Two types of braided nylon netting, Ultra Cross knotless netting and braided knotted netting, are commonly used in modern purse seines due to their good physical characteristics: high strength, low distortion or slippage, and high abrasion resistance [2, 3]. Japanese purse seiners prefer knotless nylon netting for faster sinking during shooting and less deck space for stowing. The four-strand braid construction of Ultra Cross developed by Nichimo company can produce a positive lock at strand intersections, which eliminate mesh slippage and distortion at the intersections, two main weakness of knotless netting used for fishing. The braided knotted nylon netting is commonly used by both Korean and Chinese purse seiners due to their ease of mending [2]. In addition, the braided knotted netting also has the advantage of higher breaking strength and better elasticity compared with the knotless netting. Whatever knot types is used in purse seine fisheries, it is necessary to better understand the hydrodynamic performance of netting panels with different knot types in order to estimate the static and dynamic behavior of the purse seine. The reliable drag resistance estimation is also important in experimental and numerical studies.

Numerous studies have been carried out over the past few decades by different researchers to develop empirical models of hydrodynamic response of different kinds of netting. Tsukrov et al. [4] proposed a finite element model for simulating response of a netting panel to drag forces based on Morison’s equation. In this model, the drag coefficient has different expression formulae in different range of Reynolds number. Zhan et al. [5] reported that the drag coefficient was determined by solidity and current speed. Balash et al. [6] proposed a correction formula for drag coefficient. Hosseini et al. [7] obtained drag and lift coefficients of nylon knotted netting from flume experiments to simulate the sinking behavior of a purse seine, and found that hydrodynamic coefficients depended on Reynolds number and attack angle. Kumazawa et al. [8] evaluated hydrodynamic characteristics of knotless netting panels made of three materials (Dyneema, polyamide, and polyvinyl) through flume tank experiments, and found that the drag coefficient depended on Reynolds number based on mean hydraulic depth and solidity ratio.

In general, combined effects of twine material, knot type, structural pattern, surface roughness and strand arrangement and other factors of netting attributes determine hydrodynamic characteristics of netting. However, previous studies only documented Reynolds number, solidity ratio, and attack angle as factors affecting the hydrodynamic coefficient [4–8].

We hypothesize that knot type and twine material can also have an effect on hydrodynamic coefficients. We therefore carried out a flume tank study to determine hydrodynamic coefficients of PA netting panels with different knot types (knotted and knotless) and knotless netting panels made of PA and PE. The results will help understand gear performance (e.g., sinking velocity) of tuna purse seines made of different netting panels under various fishing conditions.

## Materials and methods

### Experimental netting panels

**Fig 1:**
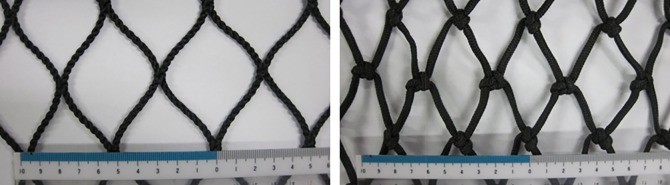
Experimental samples of knotless nylon (polyacrylamide or PA) netting (left, four-strand braid) and knotted nylon netting (right, single English knot).

Six knotless PA netting panels, two knotted nylon netting panels and one knotless polyethylene (PE) netting were tested during this study. Their structure parameters are showed in Table 1. The knotless PE netting had the exact same weave pattern and solidity ratio as net-6 nylon netting panel, and thus can be used for comparison of result on the effect of material. Mesh size and diameter were measured by taking 10 sample measurements of twine diameter and bar length with a pair of digital calipers (Nichimo Co., Ltd. Japan; resolution of 0.01 mm). All of netting panels were fabricated by Nichimo Co. (Japan). The weave pattern and structure of the four-strand knotless netting and braided knotted netting are shown in Fig 1.

**Table 1:** Structure parameters of the netting panels used in this study arranged according to their solidity ratio.

Netting No.	Twine materials	Knot types	Bar diameter (mm)	Bar length (mm)	Mesh opening angle (°)	Solidity ratio
**net-1**	nylon	knotless	1.96	52.87	45	0.073
**net-2**	nylon	knotless	1.95	46.97	45	0.081
**net-3**	nylon	knotless	3.17	46.87	45	0.132
**net-4**	nylon	knotless	3.65	48.40	45	0.147
**net-5**	nylon	knotless	3.22	48.47	30	0.155
**net-6**	nylon	knotless	4.47	45.40	45	0.187
**net-7**	nylon	knotted	3.62	45.00	45	0.168
**net-8**	nylon	knotted	3.66	43.13	45	0.177
**net-9**	polyethylene	knotless	3.74	37.93	45	0.187

### Solidity ratio of the netting

The projected area of the netting is an important factor in estimating the hydrodynamic coefficients of netting. For a netting with square mesh, the projected area is calculated using [1, 3]:

$$S_{P}=(4ld-2d^{2})\times m\times n\;\mathrm{for}\;a\;\mathrm{knotless}\;\mathrm{netting}$$ (1)

$$S_{P}=(4ld+2d^{2})\times m\times n\;\mathrm{for}\;a\;\mathrm{knotted}\;\mathrm{netting}$$ (2)

where, *m* and *n* are number of meshes in the horizontal direction (T-direction) and the vertical direction (N-direction), *d* is the twine diameter, and *l* is the bar length.

Solidity ratio (*α*) is commonly used to characterize the geometrical characteristic of netting, and is defined as the ratio of projected area (*S*_*P*_) to the total area enclosed by the outline of the netting panel (*S*_*0*_) [4, 9] as given by the following formulae:

$$\alpha =\frac{d(2l\pm d)}{l^{2}\sin\;2\varphi }$$ (3)

where, *φ* is the mesh opening angle in T-direction, and using “+” for knotted netting, and “-” for knotless netting. *φ* was maintained at 45° in all trials in our experiment, except for net-5 where *φ* was 30° (Table 1).

### Hydrodynamic coefficients

The dimensionless coefficients *C*_*D*_ (drag coefficient) and *C*_*L*_ (lift coefficient) of plane netting were calculated using following expressions [8, 10]:

$$C_{D}=\frac{2F_{D}}{\rho \alpha S_{0}V^{2}}$$ (4)

$$C_{L}=\frac{2F_{L}}{\rho \alpha S_{0}V^{2}}$$ (5)

where *F*_*D*_ and *F*_*L*_ are the measured drag and lift forces acting on the netting panel; *ρ* is density of water; *V* is flow velocity; *α* is solidity ratio; *S*_0_ is the outline area of netting panel. *α S*_0_ represents the projected area of the netting panel.

### Experimental processes

**Fig 2:**
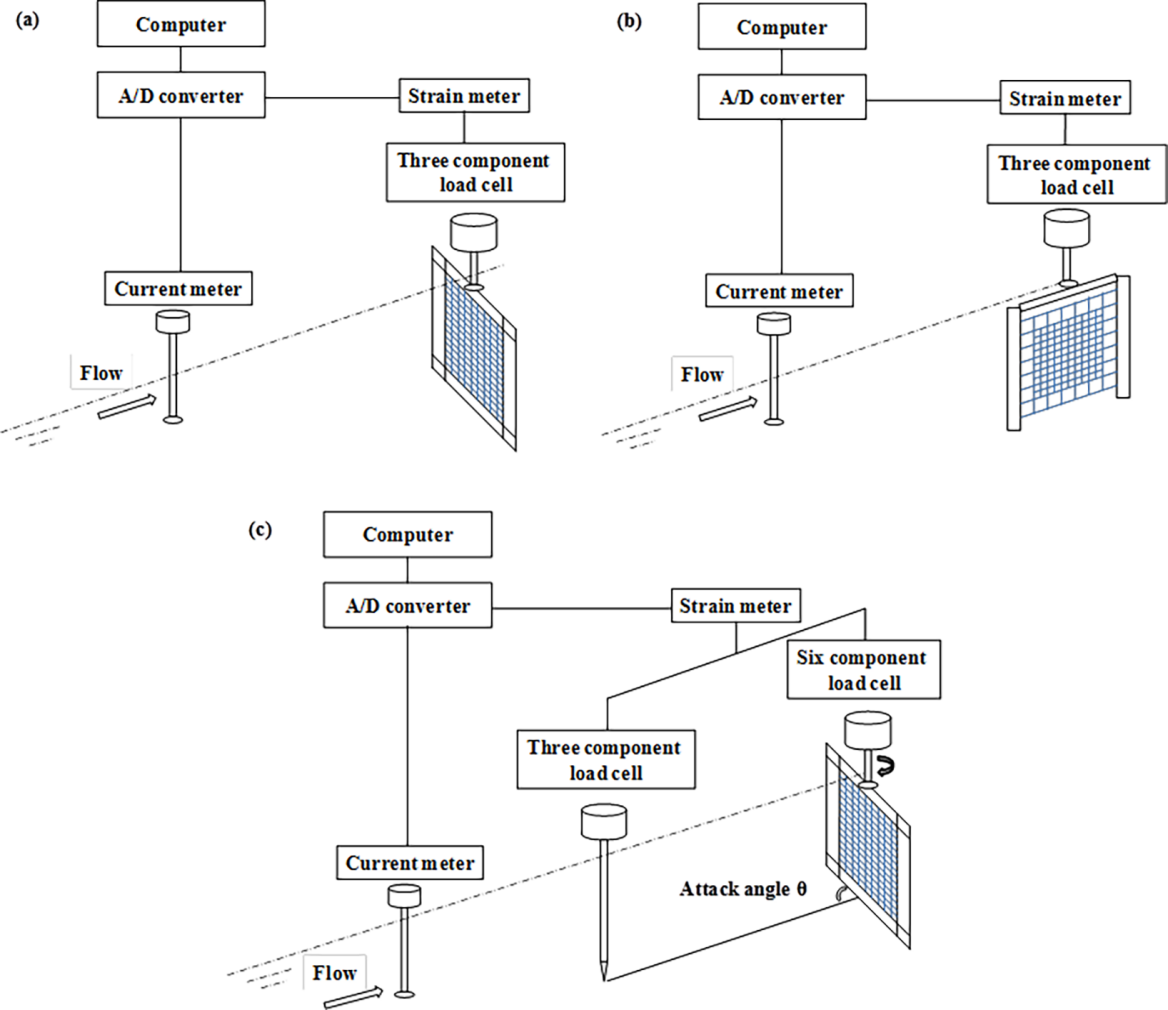
Diagrammatic sketch of experiment setup and apparatus. (a) normal to the flow, (b) parallel to the flow, (c) an angle to the flow.

The experiment was carried out in a flume tank at the Tokyo University of Marine Science and Technology (TUMST). Fig 2 illustrates experimental rigging. The flume tank measured 9.0 m in length, 2.2 m in width, and 1.6 m in depth. The dimension of all test netting samples was 50 cm × 50 cm. Two types of frame were employed to test netting panels in different flow directions (Fig 2). To avoid wake turbulence, two streamlined frames (normal to flow: 70 cm × 70 cm; parallel to flow: 100 cm × 60 cm) were used to investigate steady load on plane netting positioned at 90° or 0° to the incoming flow. For the netting panel inclined to incoming flow, a 50 cm × 50 cm frame made of cylindrical stainless steel was used to measure the hydrodynamic forces of netting panels under different attack angles. The advantage of a cylindrical frame is that it can present a steady drag at different attack angles ranging from 0° to 90°. In order to compensate for the frame resistance, tests were also conducted for frames without a netting panel.

When testing netting panels set at normal (90°) or parallel (0°) to the flow, the streamlined frame was attached to a three component load cell (Electronic industrial Co., Ltd., maximum load 49 N) and without pre-tension to prevent vibration of the frame (Fig 2A, Fig 2B). The flow velocity was measured using a propeller tachometer located at 1.2 m in front of the frame, with the flow velocity ranging from 40 cm/s to 130 cm/s, and incremented at 10 cm/s intervals.

At higher flow velocity, shockwaves develop on the cylindrical frame, interrupting the flow of water and affecting the measured result when the netting panel inclined to the flow. To overcome this, we pre-tensioned the cylindrical frame with a steel cable (8 mm diameter) which was connected to a cylinder placed 150 cm ahead of the test frame (Fig 2C). We then used two load cells to obtain forces on the pre-tension cylinder and on the test frame. The flow velocity was between 40 and 120 cm/s with 20 cm/s increments, and the attack angle was changed from 10° to 80° at 10° intervals.

The water temperature was maintained at 12°C during the experiment. Data for each experiment were gathered at 20 Hz over a period of 20 s using a data acquisition system [3]. The values of hydrodynamic force and flow velocity were passed through a digital voltmeter, and recorded in a computer. The data acquisition is described by the following: First, the voltage signals of drag force and lift force are transmitted to the strain meter by means of the sensor when the flow thought the netting and frame; Second, the strain meter amplify and integrate the signals from all the sensors, producing analog signals information that can be displayed continuously online; Third, the A/D converter changed analog signals to digital signals; 5) Final, the measured forces are figured by calibration coefficients on computer. Through the above mentioned way, the flow velocity signals also are obtained from propeller tachometer sensor.

Six knotless nylon netting samples with different solidity ratios were tested to derive an empirical formula for the knotless nylon netting. The empirical formula was then used to predict hydrodynamic coefficient of knotted netting with the same solidity ratio. The differences in hydrodynamic coefficients between knotted and knotless netting were then compared. The hydrodynamic characteristics of PA netting and PE netting was also compared to understand differences between netting materials.

## Results

### Drag coefficient when the netting is normal to the flow

**Fig 3:**
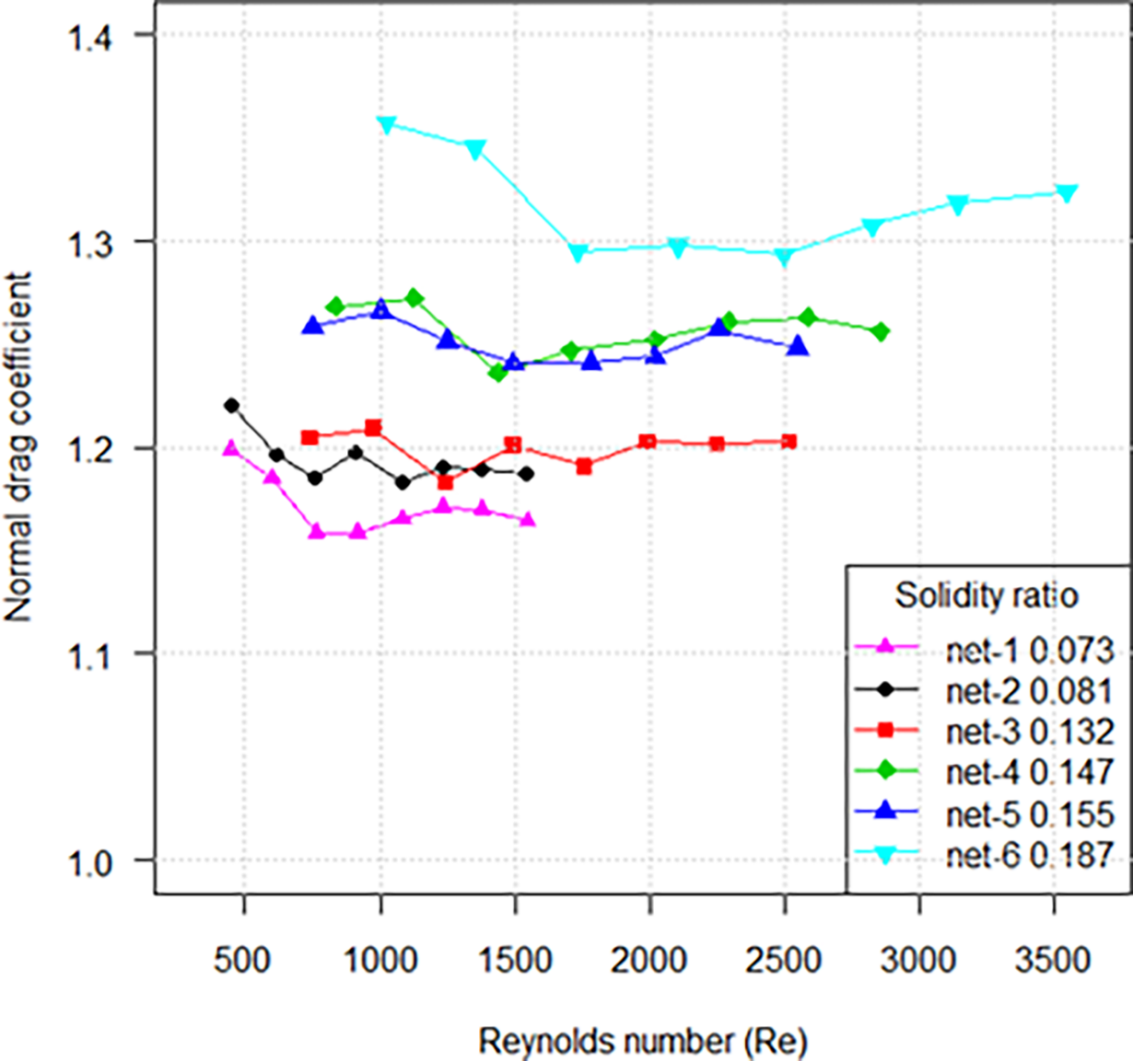
Relationships between drag coefficient of knotless nylon netting with different solidity ratios and Reynolds number when the netting was set normal to the flow.

The drag coefficient when the netting is normal to the flow (*C*_*D*90_) of knotless nylon netting panels with different solidity ratios as a function of Reynolds number (Re = *Vd*/*v*, *v* is dynamic viscosity of water) based on twine diameter is shown in Fig 3. The normal drag coefficient decreased as Reynolds number increased, and the netting with higher solidity ratio showed a greater drag coefficient. Nonlinear regression on the data produced the following formulae for knotless netting panels with different solidity ratios:

CD90=1.691α0.114Re−0.011(400<Re<3600) (6)

**Fig 4:**
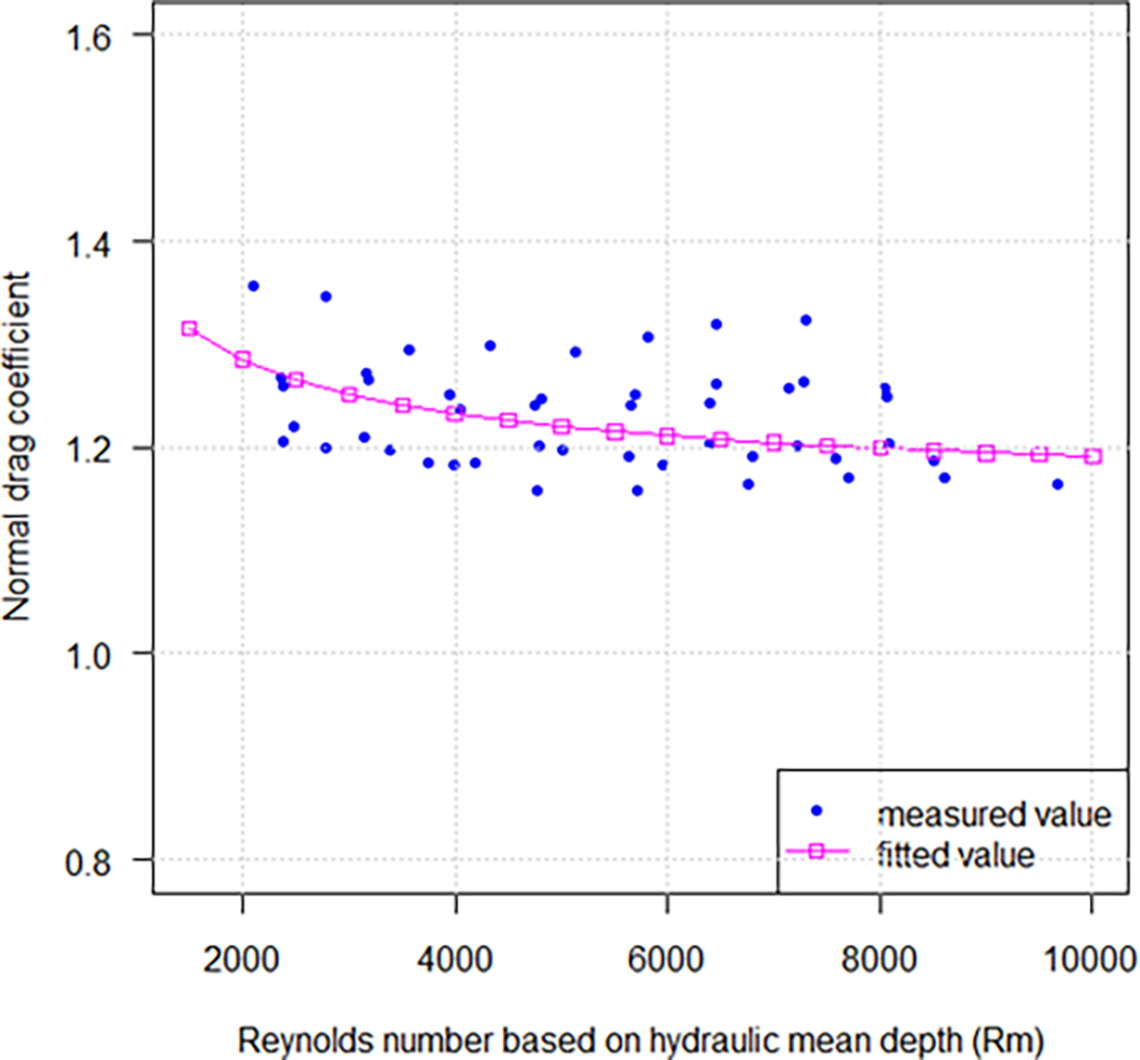
Relationships between drag coefficient of nylon netting with and *R*_*h*_ when setting normal to the flow.

The characteristic length can be represented by hydraulic mean depth (*h*) in case of netting panel normal to the flow. For diamond mesh, the hydraulic mean depth, *h* = 0.25 *βl* sin 2*φ* (*β* = 1−*α*, *β*, is porosity of netting), thus, *R*_*h*_ = *hV*/*v*. Fig 4 illustrates that, with the increasing Reynolds number *R*_*h*_ based on hydraulic mean depth as the characteristic length, the normal drag coefficient decreased and approached a steady valus of about 1.2. So, for a knotless nylon netting mesh geometry subjected to a flow 30< *V* <120 cm/s, *R*_*h*_ ranges from 1.5×10^3^ to 1.0×10^4^ and *C*_*D*90_ is given by the following expression:

$$C_{D90}=19.371R_{h}{}^{-0.643}+1.139$$ (7)

**Fig 5:**
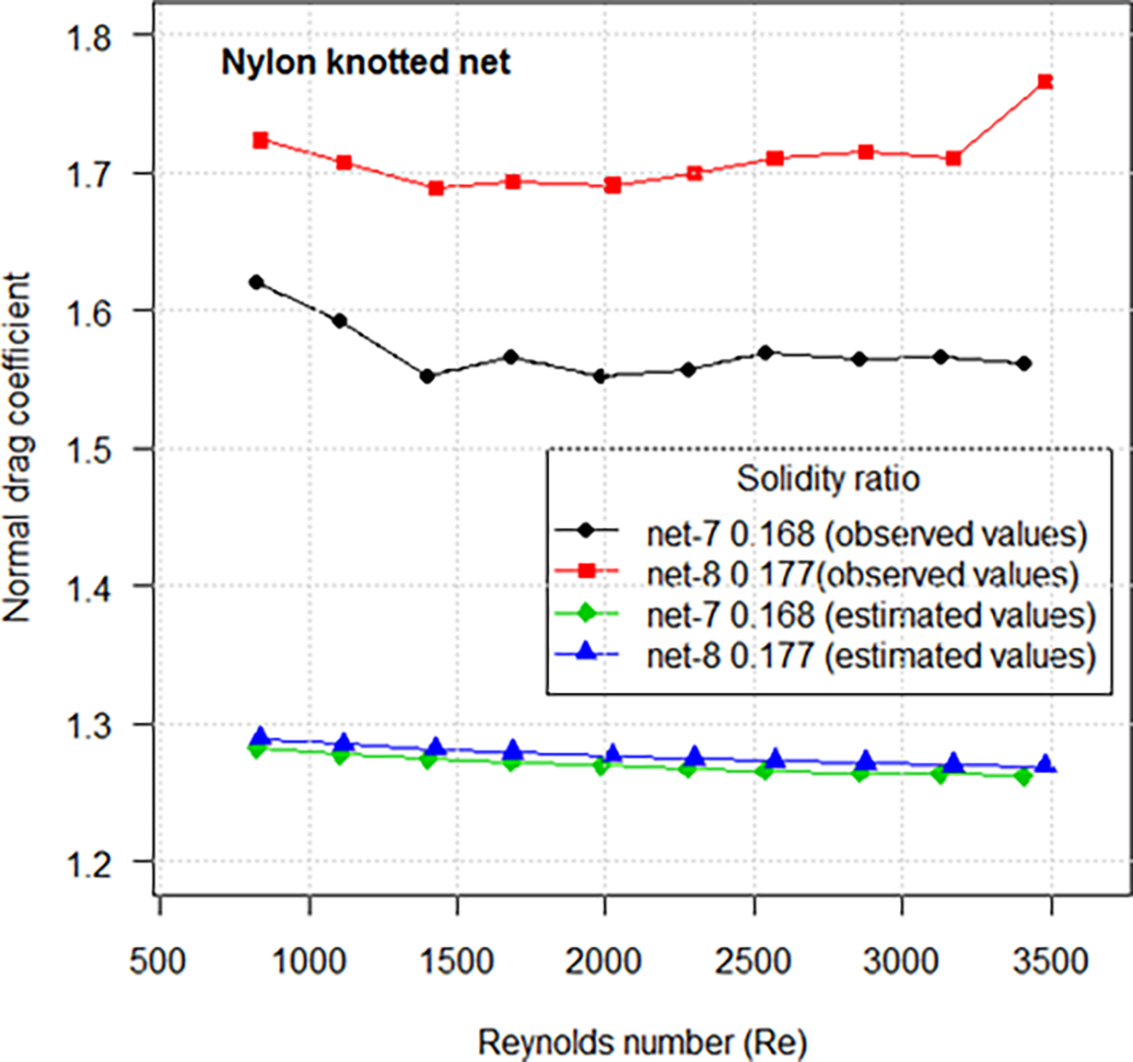
Comparison of drag coefficients when the netting is normal to the flow between knotless netting panels and knotted netting panels.

The nylon netting with higher solidity ratio demonstrates a greater *C*_*D*90_ both for the knotless netting and for the knotted netting (Fig 5). The estimated *C*_*D*90_ by fitted equation makes up approximately 74–81% of observed values. Thus, we estimated the *C*_*D*90_ generated by knot accounted for 19–26% of total drag coefficient by extrapolation from experiment results (Fig 5).

### Drag coefficient when netting panel is parallel to the flow

**Fig 6:**
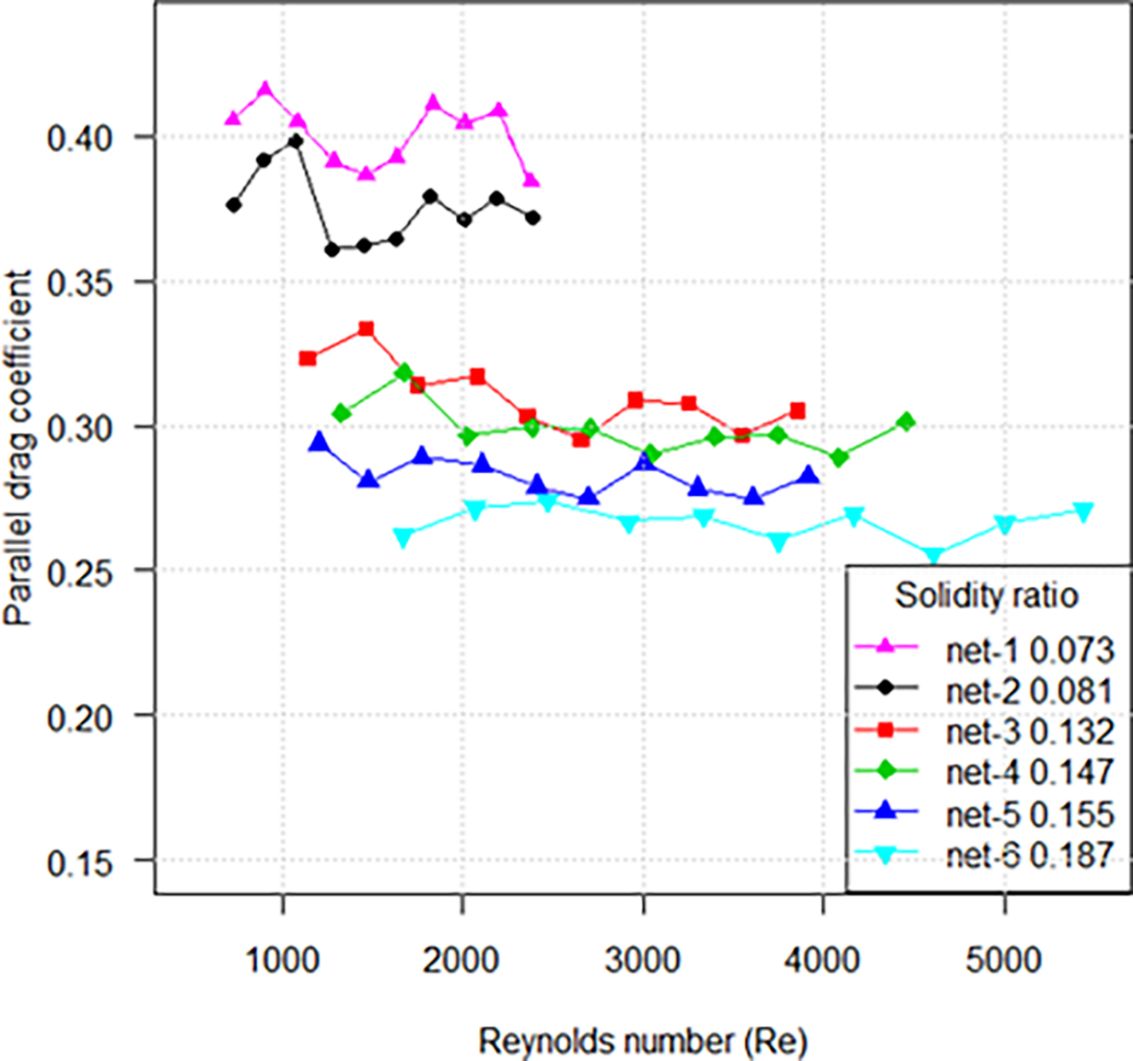
Drag coefficient for netting parallel to the flow as a function of Reynolds number for various solidity ratios.

The drag coefficients when the netting is parallel to the flow (*C*_*D*0_) of nylon knotless netting panels are shown in Fig 6. The drag coefficient decreased as Reynolds number based on twine diameter increased. The sharp change of *C*_*D*0_ mainly confined to small solidity ratio when Reynolds number was over 2000, while the drag coefficient with greater solidity ratio remained relatively stable. In general, the drag coefficient of nylon netting with lower solidity ratio was greater than one with higher solidity ratio. The drag coefficient of knotless nylon netting when setting parallel to the flow can be expressed as the function of both Reynolds number and solidity ratio in the following form:

CD0=0.172α−0.407Re−0.031(500<Re<5500) (8)

**Fig 7:**
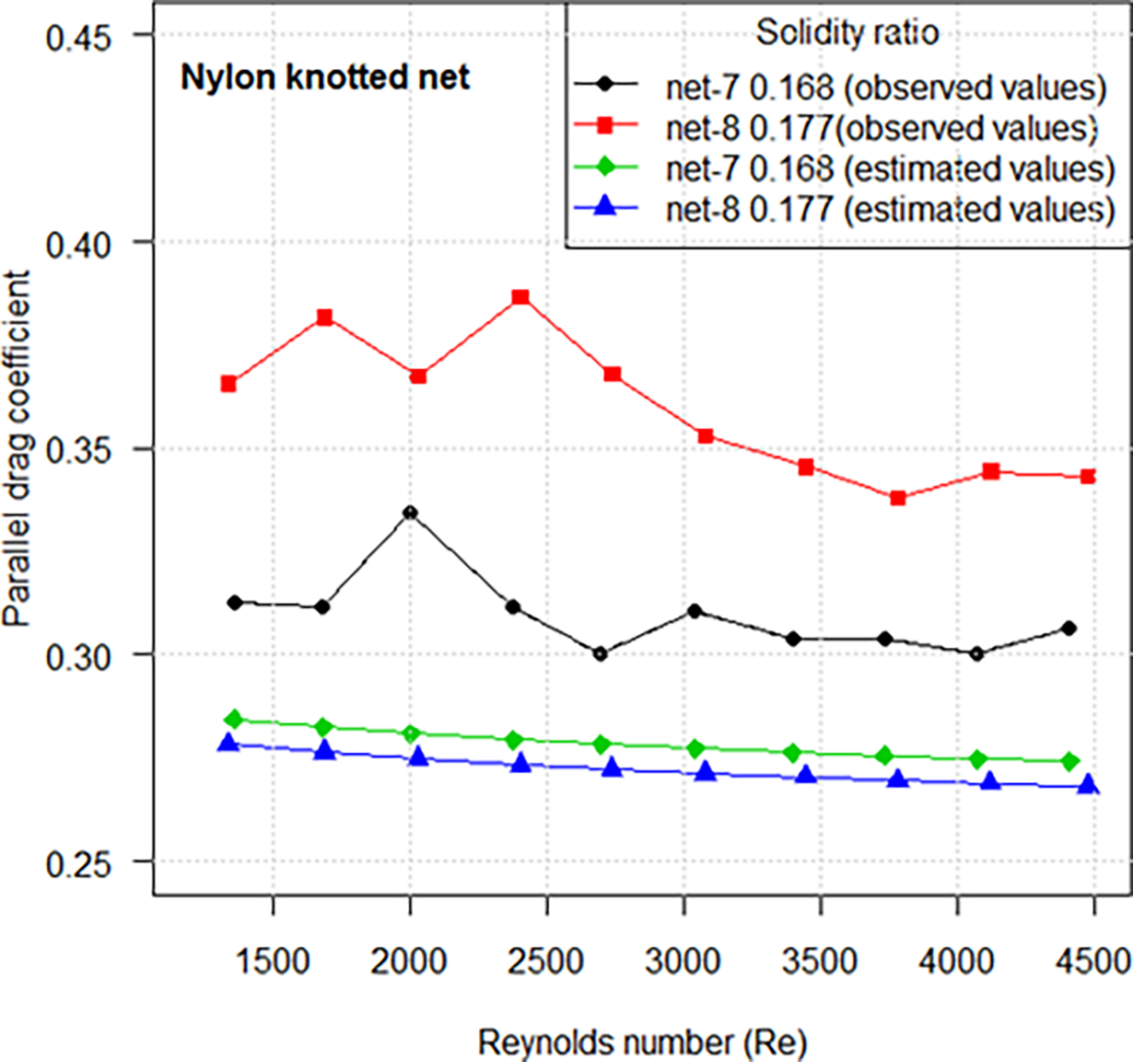
Comparison of parallel drag coefficients between knotless netting panels and knotted netting panels.

The *C*_*D*0_ of knotted netting with higher solidity ratio exhibit a greater coefficient, which is opposite to the results of knotless netting (Fig 7). The estimated *C*_*D*0_ makes up approximately 76–90% of observed values. We thus estimated the *C*_*D*0_ generated by knots accounted for 10–24% of total drag coefficient by extrapolation from experiment results.

### Drag and lift coefficient for netting panel inclined to the flow

**Fig 8:**
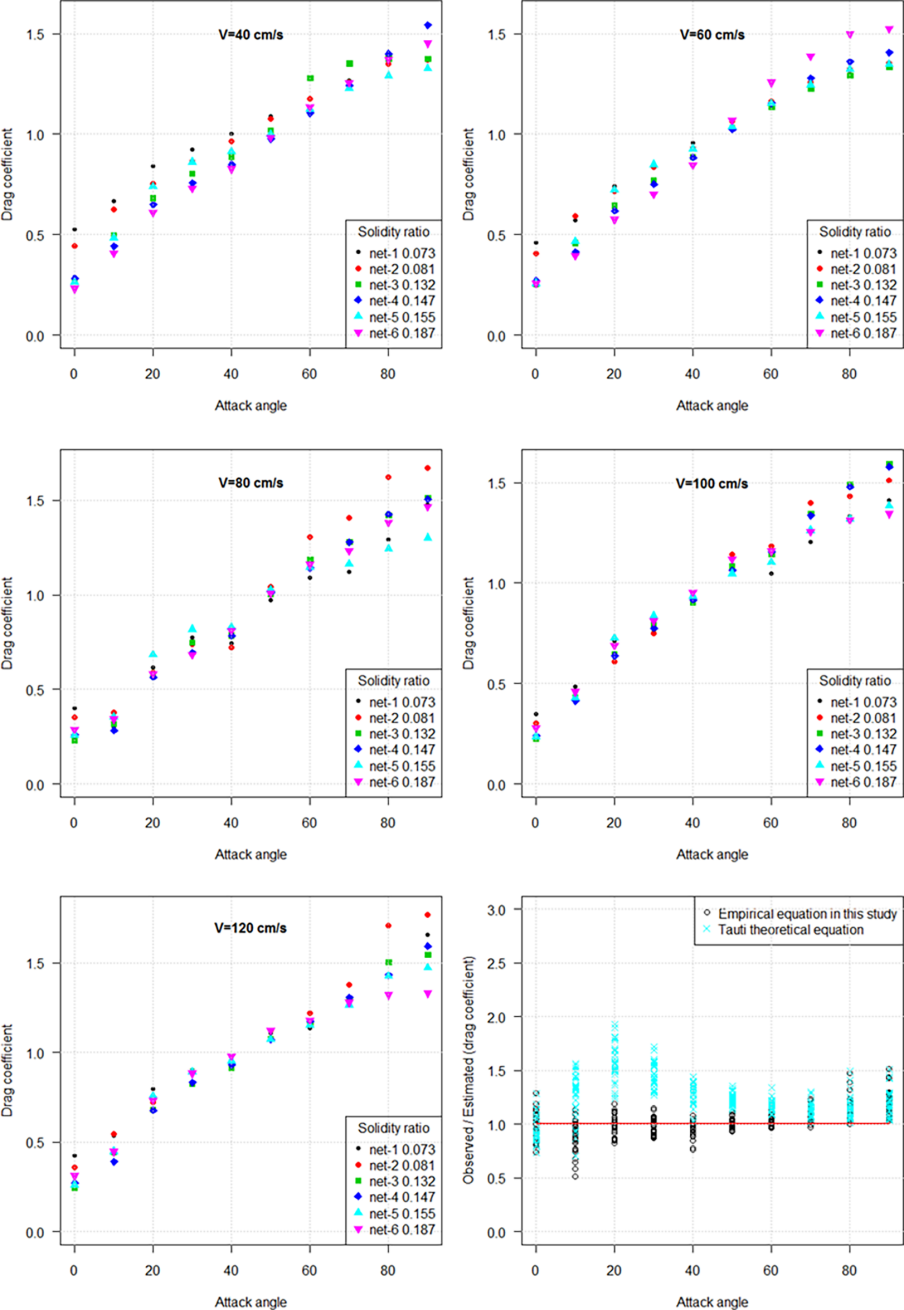
Comparison of drag coefficient of inclined nylon netting panels with varied solidity ratios against attack angle.

Fig 8 shows that the inclined drag coefficient of knotless PA netting panel increased with the increase of attack angle (*θ*). The inclined drag coefficient (*C*_*Dθ*_) decreased as solidity ratio increased when attack angle was less than 50°, but increased as solidity increased at attack angles from 50° to 90°. Hence the solidity ratio had a dual influence on drag coefficient when the netting was set inclined to the flow.

**Fig 9:**
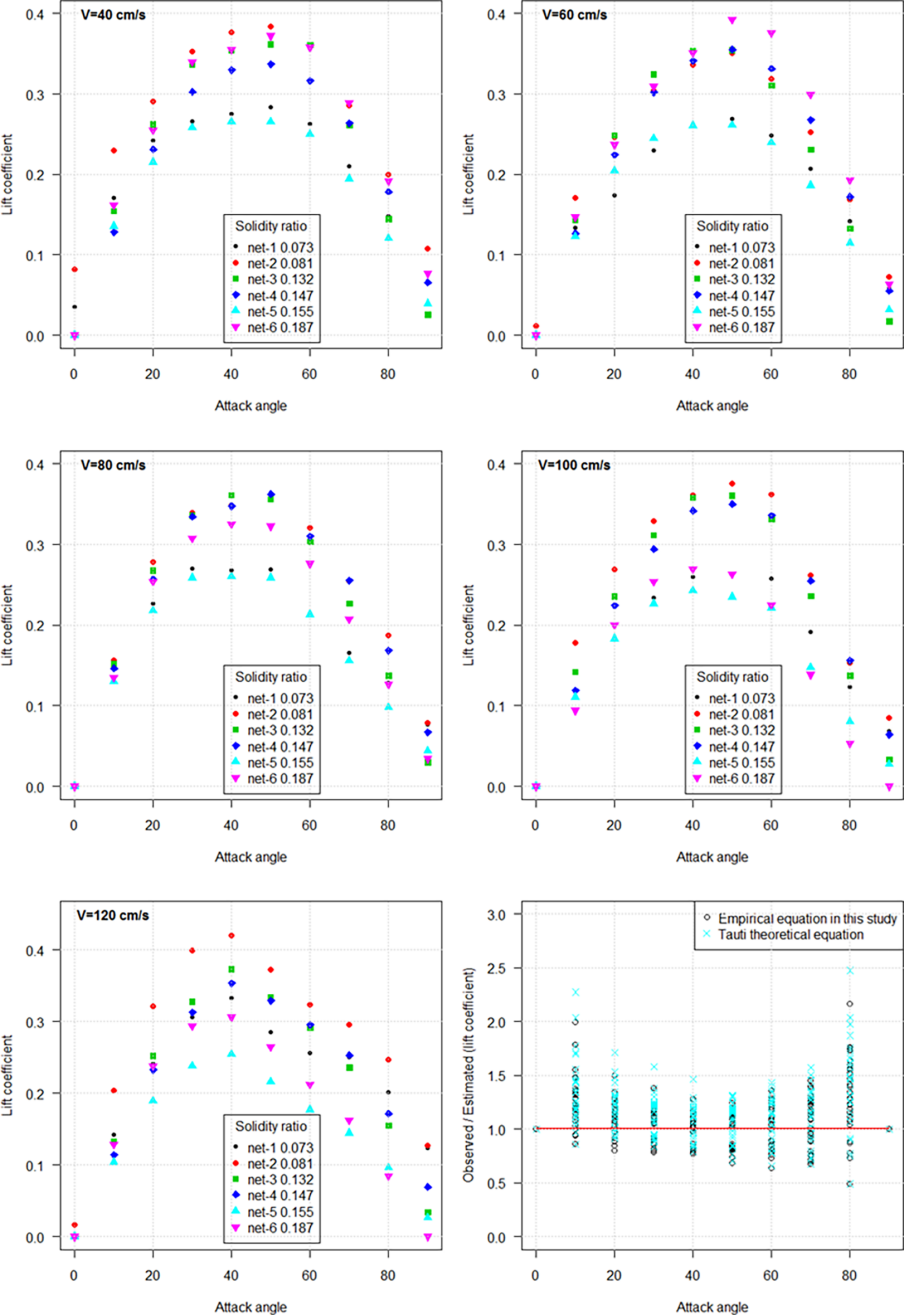
Comparison of lift coefficient of inclined knotless nylon netting panels with varied solidity ratios against attack angle.

The lift coefficient (*C*_*Lθ*_) reached a culminating point at an attack angle of 50° and subsequently decreased with the increased attack angle (Fig 9). Both the drag and lift coefficients increased as flow velocity decreased.

We deduced the following formula for the drag coefficient of knotless nylon netting based on Tauti’s theoretical formula and Kumazawa et al. [8] formula:

$$C_{D\theta }=C_{D90}\sin\theta +(C_{D0}-0.889\alpha \sin\theta )\cos^{2}\theta$$ (9)

Based on Tauti’s theoretical formula, the inclined lift coefficient formula was proposed:

$$C_{L\theta }=0.38\alpha^{-0.162}C_{D90}\sin\theta \cos\theta$$ (10)

**Fig 10:**
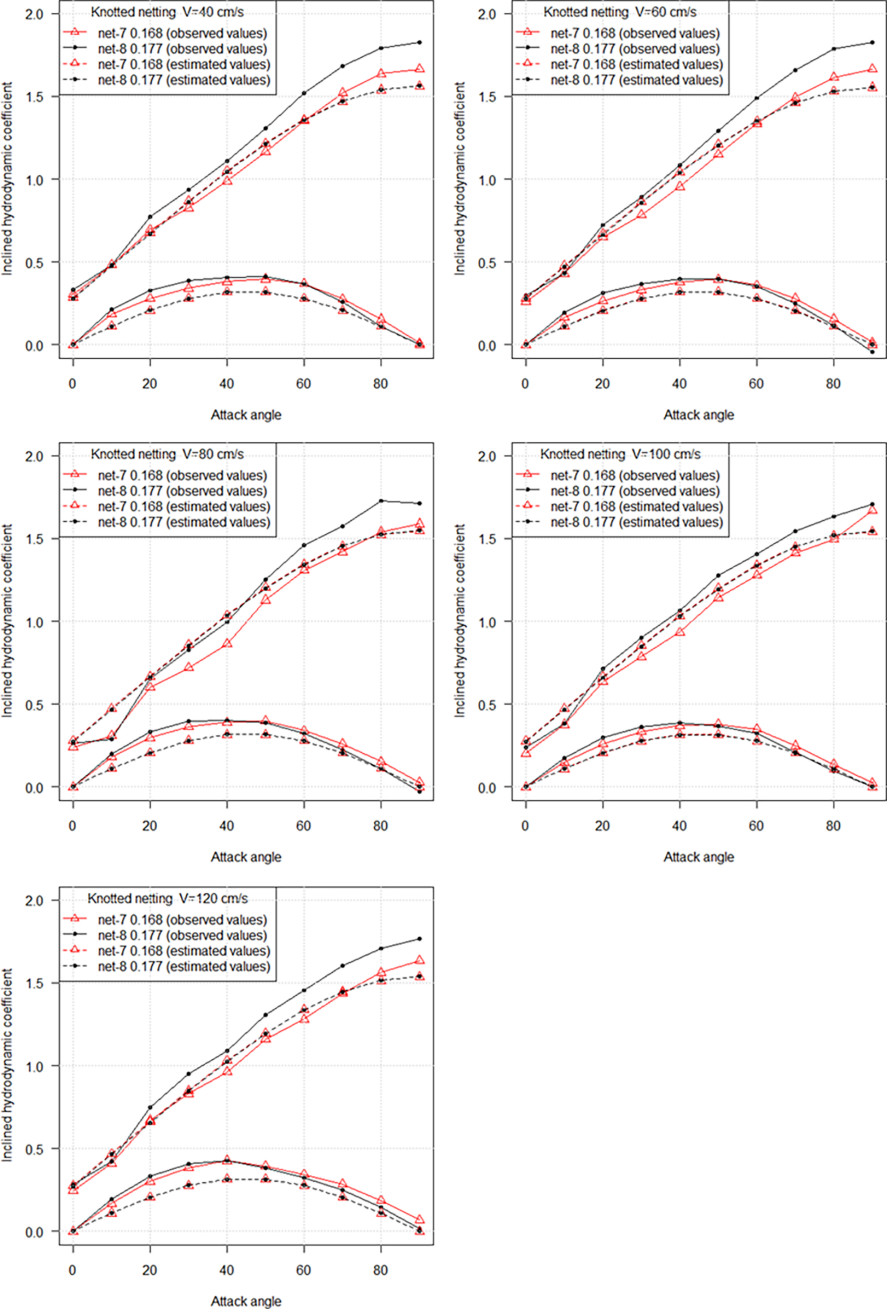
Comparison of inclined hydrodynamic coefficients between knotless netting panels and knotted netting panels at various attack angles and flow velocities.

The comparison of inclined coefficients between knotted netting and knotless netting at various attack angles and flow velocities is shown in Fig 10. The estimated values correspond to hydrodynamic coefficient of knotless netting with the same solidity ratio as knotted netting. For knotted nylon netting, the inclined drag coefficient increased as attack angle increased, the lift coefficient reached a culminating point at an attack angle of 50° and subsequently decreased as the attack angle increased to 90°, similar to knotless netting. It was found that there was remarkable difference in drag coefficients between knotted netting and knotless netting, particularly when attack angles ranged from 50° to 90° (Fig 10). But there existed remarkable difference in lift coefficient between different netting types when attack angle was less than 50°. It could be inferred from these results that the differences were due to the different knot types.

### Hydrodynamic characteristic comparison of the PA netting and PE netting

**Fig 11:**
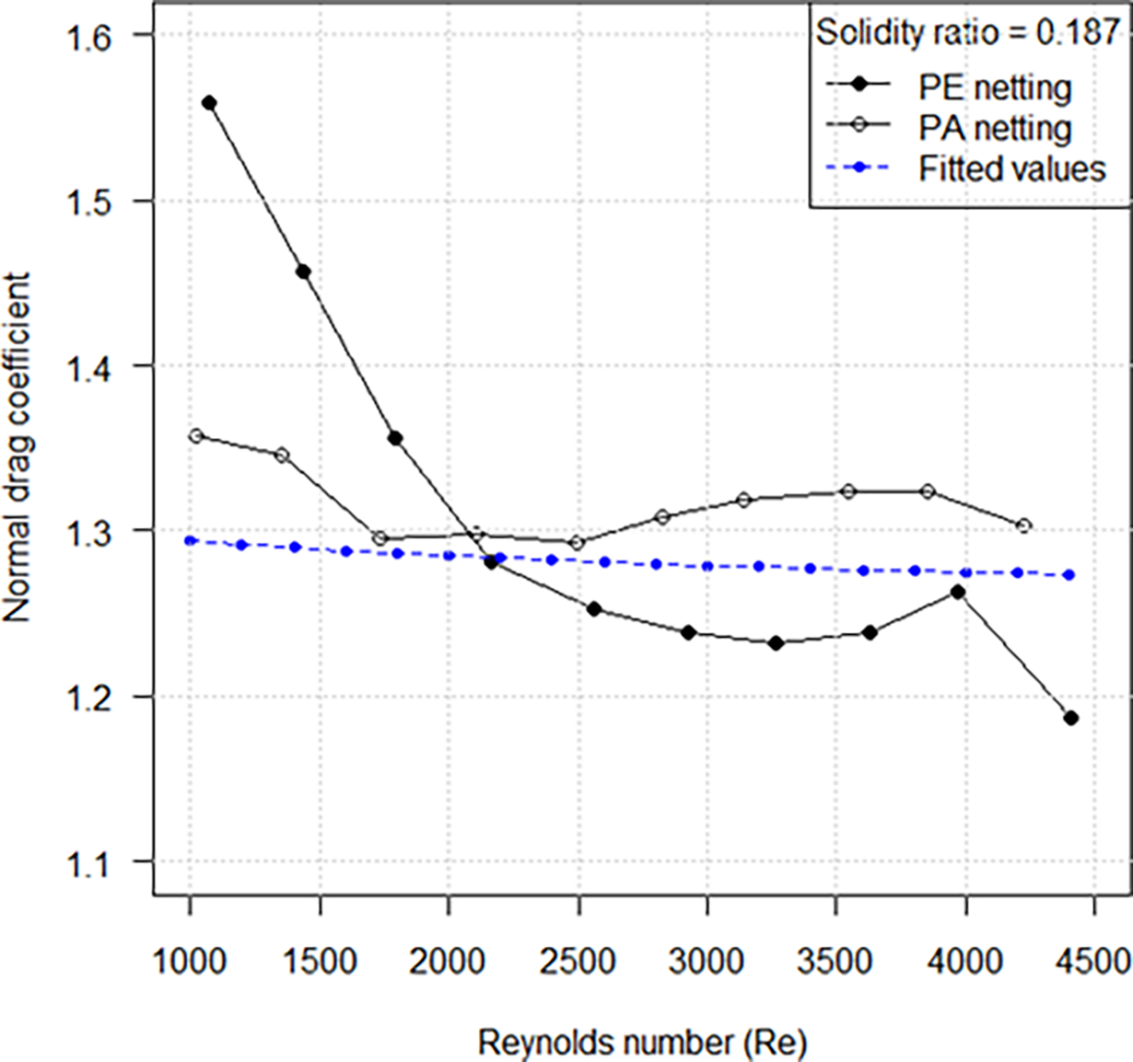
Comparison of drag coefficients between knotless nylon (PA) netting and knotless polyethylene (PE) netting.

For same weave pattern (four-strands braided) and solidity ratio, the drag coefficients of knotless PE netting were dominant at low Reynolds number (Re < 2200), but the drag coefficients of PA netting were greater than that of PE netting and stayed approximately constant up to 1.3 when Reynolds number was larger than 2200 (Fig 11). To obtain the values of drag coefficients representative for the entire tested range of Reynolds number, the fitted regression curves by Eq 6 presented in Fig 11 were utilized. It was found that the drag coefficients of PA netting were underestimated by about 1–5%. But the fitted regression for PA netting cannot reliably predict the drag coefficients of PE netting. This also verified the differences in hydrodynamic characteristic between PA netting and PE netting.

## Discussion

In this study, the hydraulic mean depth (*h*) defined as the ratio of area to the perimeter of the mesh [11] was used as characteristic length for netting when it was set normal to the flow. We suggested that the flow through the plane netting could be considered as a flow in the pipe, the hydraulic mean depth can thus be used as the characteristic length to determine the range of Reynolds number in experiment. Considering the porosity of netting, the characteristic length of experimental netting was characterized by a combination of the hydraulic mean depth and porosity, *h* = 0.25 *βl* sin 2*φ*. A better fitting prediction result was presented in Fig 4. Our findings show that the drag coefficient decreased and approached a steady value distributed in a space between 1.2 and 1.4 when *R*_*h*_ ranged from 1.5×10^3^ to 1.0×10^4^, similar to that reported by Miyazaki and Takahashi [11].

### Comparative analysis with previous studies in normal drag coefficients

**Fig 12:**
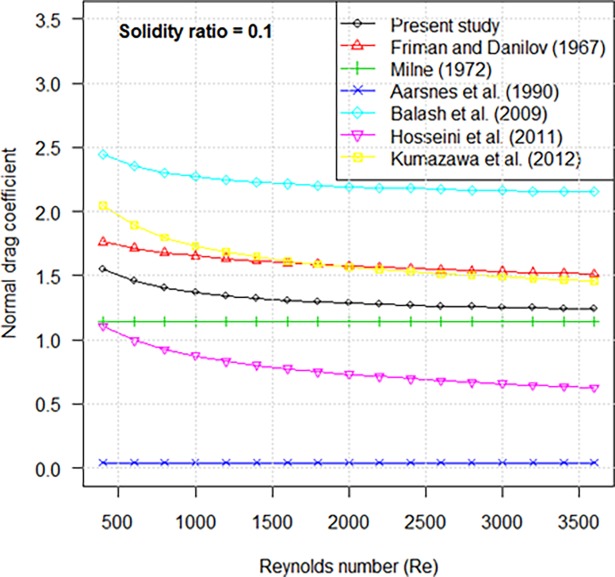
Comparison of normal drag coefficients vs. Reynolds number.

Fig 12 shows the drag coefficients of knotless nylon netting panels together with drag coefficients predicted by previous researchers. For drag coefficient when the netting is normal to the flow, the empirical formulae from Milne [12] and Aarsnes et al. [13] only depended on solidity ratio, thus the two constants were shown in Fig 12 under different ranges of Reynolds number. The formulae proposed by Fridman and Danilov [14], Balash et al. [6], and Kumazawa et al. [8] were related to solidity ratio and Reynolds number, but the predicted drag coefficients were greater than the results of this study. But the formula developed by Hosseini et al. [7] as a function of Reynolds number resulted in lower predictions than our results. These differences may be attributable to the material and solidity ratio of the experimental netting we used and conditions of our experiment setup. For example, Balash’s formula used the circular cylinder drag coefficient [6]; Hosseini’s experimental netting was nylon braided knotted netting with a smaller solidity ratio [7]. In general, we recommend that researcher use the empirical formulae of this study to predict hydrodynamic coefficients of nylon netting when placed normal to incoming flow.

### Comparative analysis with previous studies in parallel drag coefficients

**Fig 13:**
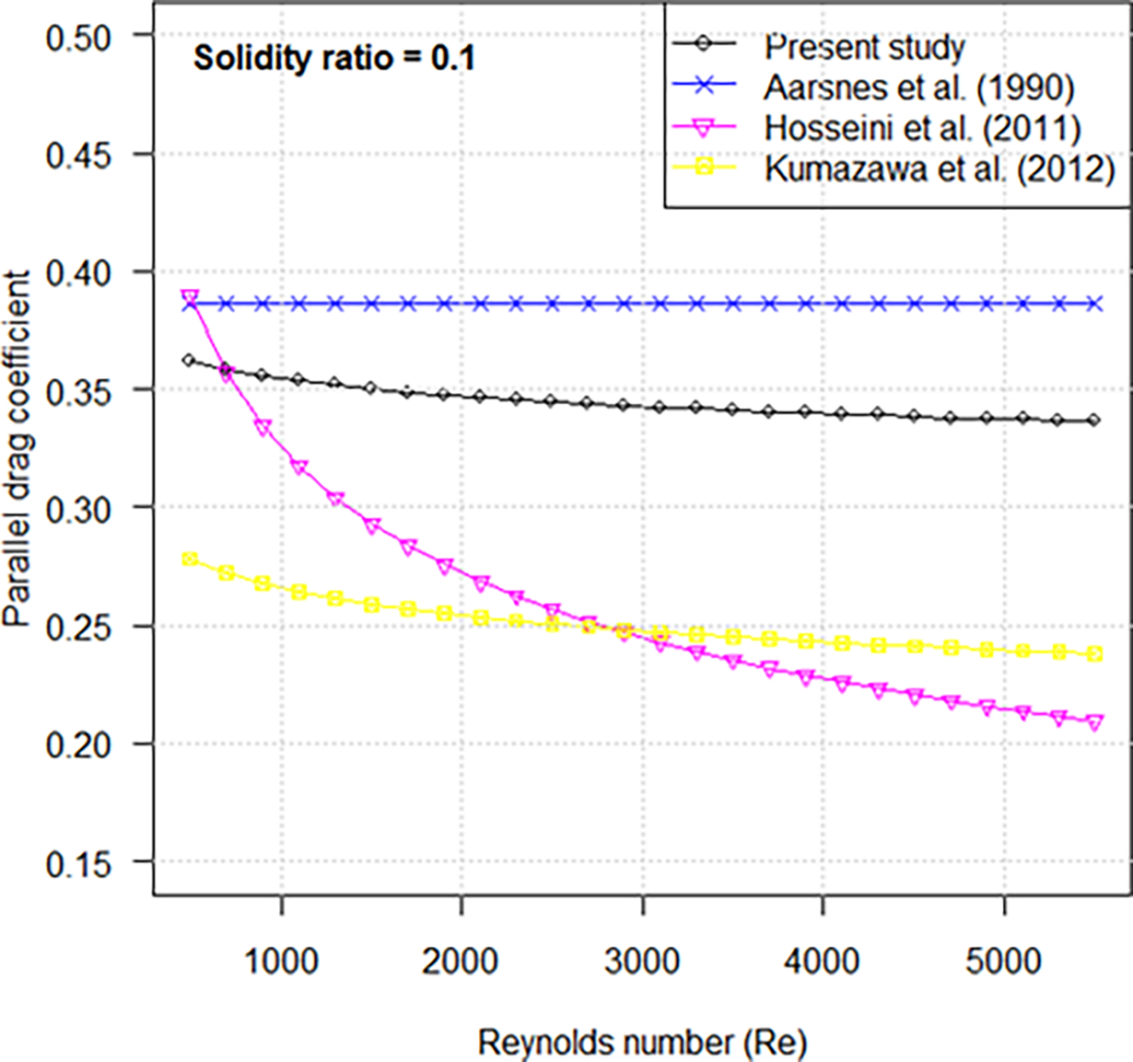
Comparison of parallel drag coefficients vs. Reynolds number.

Kumazawa et al. [8] reported that there was only a slight difference in drag coefficients when the plane nettings made of different materials were set parallel to the flow. So, we compared our parallel drag coefficient formula against previously empirical formulae for the nylon netting parallel drag coefficient. A constant coefficient reported in Aarsnes et al. [13] was slightly higher than that in this study (Fig 13). This difference can be attributed to the difference in netting solidity ratio (Aarsnes might have used the netting with lower solidity). However, the parallel drag coefficients reported in Hosseini et al. [7] and Kumazawa et al. [8] were lower than our study. This may have been caused by the difference in the experimental method, including experiment setup and testing condition. But the influence of shading effect (produced by the twine as flow moves through the netting panel) on plane netting cannot be neglected if the netting is set parallel to the flow. Under the same experiment condition, the shading effect of knotted netting is higher than that of knotless netting. However, Hosseini’s drag coefficient formulae only relate to Reynolds number [7]. The parallel drag coefficient was less than our result though his experiment samples were made of knotted nylon netting.

### Comparative analysis with previous studies in inclined hydrodynamic coefficients

**Fig 14:**
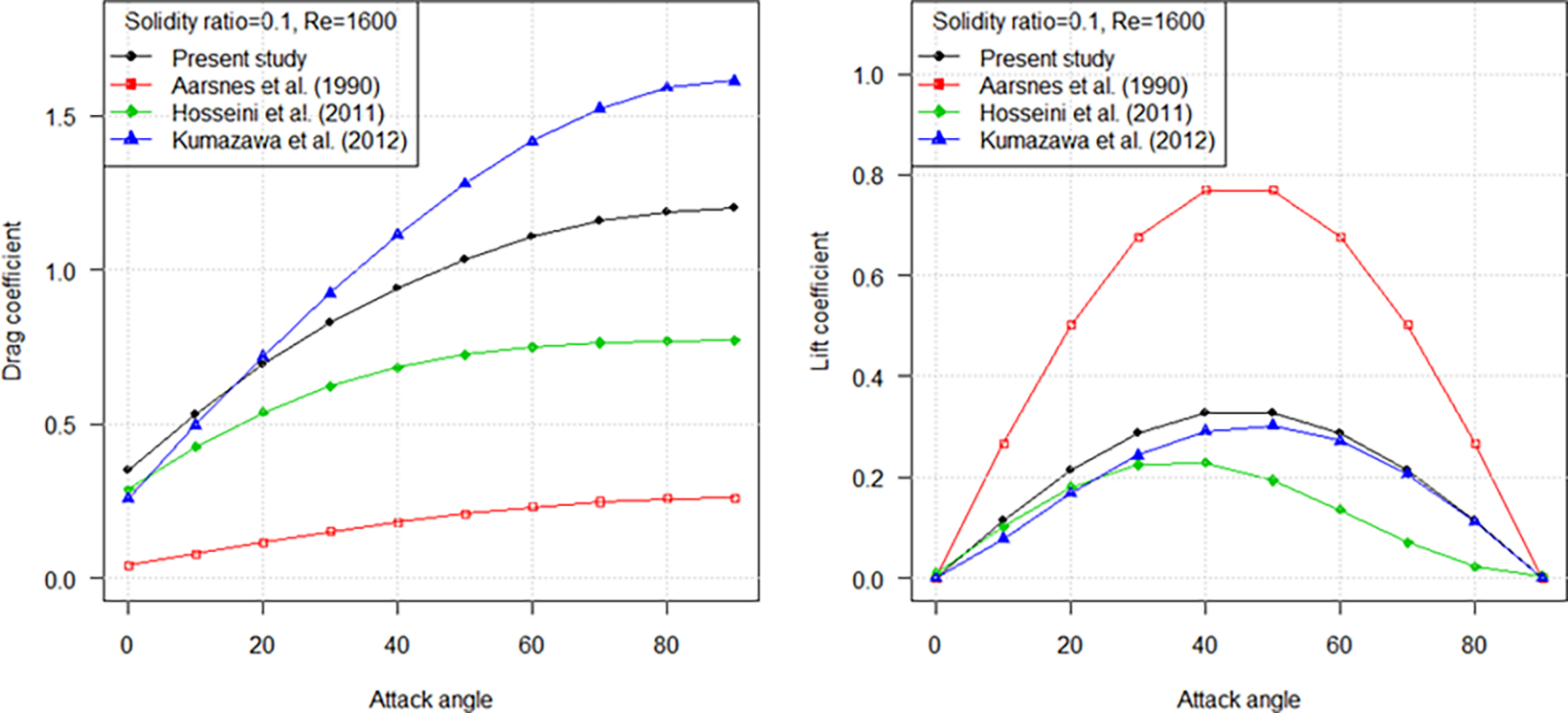
Relationship between hydrodynamic coefficients of netting panels and attack angles: Drag coefficient (left), lift coefficients (right).

In this part, a summary of available studies on drag coefficient of plane netting, both experimental and theoretical, when setting inclined to the flow was provided. Different inclined drag and lift coefficients formulae have been proposed according to the data measured during the experiment [7, 8, 13]. Fig 14 shows that the drag coefficients of inclined netting in Hosseini et al. [7] and Aarsnes et al. [13] reports are lower than those observed in this study. The inclined drag coefficient in Kumazawa et al. [8] study was slightly higher than ours. It was due to the netting samples tested in their experiment have lower solidity ratio. In general, the obtained results agree satisfactorily with those deduced by empirical formula by Kumazawa et al. [8], particularly in lift coefficient.

The hydrodynamic coefficients when the netting is inclined to the flow increased as flow velocity decreased. An increase in the projected area with increasing attack angle resulted in the increase of the drag coefficient. The decreased in both coefficients with increasing flow velocity was caused by the squared relationship between the flow velocity and coefficients (Eq (4) and Eq (5)). We also introduced the ratio of the observed *C*_*Dθ*_ to estimated *C*_*Dθ*_, and the ratio of the observed *C*_*Lθ*_ to estimated *C*_*Lθ*_, to compare the results calculated by Tauti’s theoretical equation with our estimated values (Fig 8, Fig 9). It was found that the drag and lift coefficients estimated by Tauti’s theoretical equation were less than the observed drag and lift coefficients, particularly for attack angles from 0° to 30°. However, our empirical equation calculated results matched well with the observed values.

### The effect of knot types and twine materials on hydrodynamic characteristics

The knot type is one of the most important factors that affect hydrodynamic coefficients of fishing net. We therefore, while testing the drag coefficient of nylon knotless netting, performed a parallel test of nylon knotted netting. For same solidity ratio, the average drag coefficient of knotless PA netting panel is approximately 83% of that of knotted netting. This may be attributable to the velocity reduction from the flow through netting panel [1]. Løland [15] found that the flow velocity is reduced through netting panel by: *V*_*wake*_ = *rV*_∞_, where, *V*_*wake*_ is local velocity in the wake of the netting panel and *V*_∞_ is incoming flow. Løland [15] also proposed a linear relation between the velocity reduction coefficient *r* and the drag coefficient *C*_*D*_: *r* = 1−0.46*C*_*D*_. Influenced by knot, the shading effect is most apparent when flow through a knotted netting panel, resulting in a larger drag coefficient (Fig 7). For purse seine s, it is important to use netting panels with less drag for speedy sinking. It is thus useful to know that knotless nylon nettings are chosen due to their faster sinking and easier storage, whereas the reason for choosing the knotted nylon netting is the easier to mend and the property of stronger and better elasticity. The solidity and hydrodynamic resistance may be the main factor for the difference in sinking performance between knotless and knotted nylon netting.

The hydrodynamic coefficients for each netting panel reach steady values for flow velocities of 50–60 cm/s and stay approximately constant up to 120 cm/s (maximum experiment flow velocity). This is attributed to the transition of the flow regime from viscous laminar flow at low velocities to developed turbulent flow at higher velocities [9]. This also explains why a critical point (*Re* of about 2200) is presented in Fig 11, which shows the hydrodynamic characteristic comparison of the PA netting and PE netting. For the same weave pattern, the different flow regimes have different influences on the hydrodynamic coefficients of netting with different materials. It is justified to attribute to the difference in netting materials hardness and roughness.

The drag forces on netting panels depend not only on solidity ratio but also on the ratio of turbulence region area around netting threads to the outline area of a netting panel. For the similar solidity ratio, netting panels with larger mesh size will have larger areas of undisturbed flow, resulting in lower force on netting panels, such as net-1 with mesh size of 52.87 mm and net-3 with mesh size of 46.97 mm. Many researchers have applied Computational Fluid Dynamic (CFD) or Particle Image Velocimetry (PIV) along with experiments to investigate the flow characteristics through and around netting panel [16]. This will provide an effective way to visualize flow pattern so as to explain differences in drag of different netting panels.

### The effect of experimental setup on results

**Fig 15:**
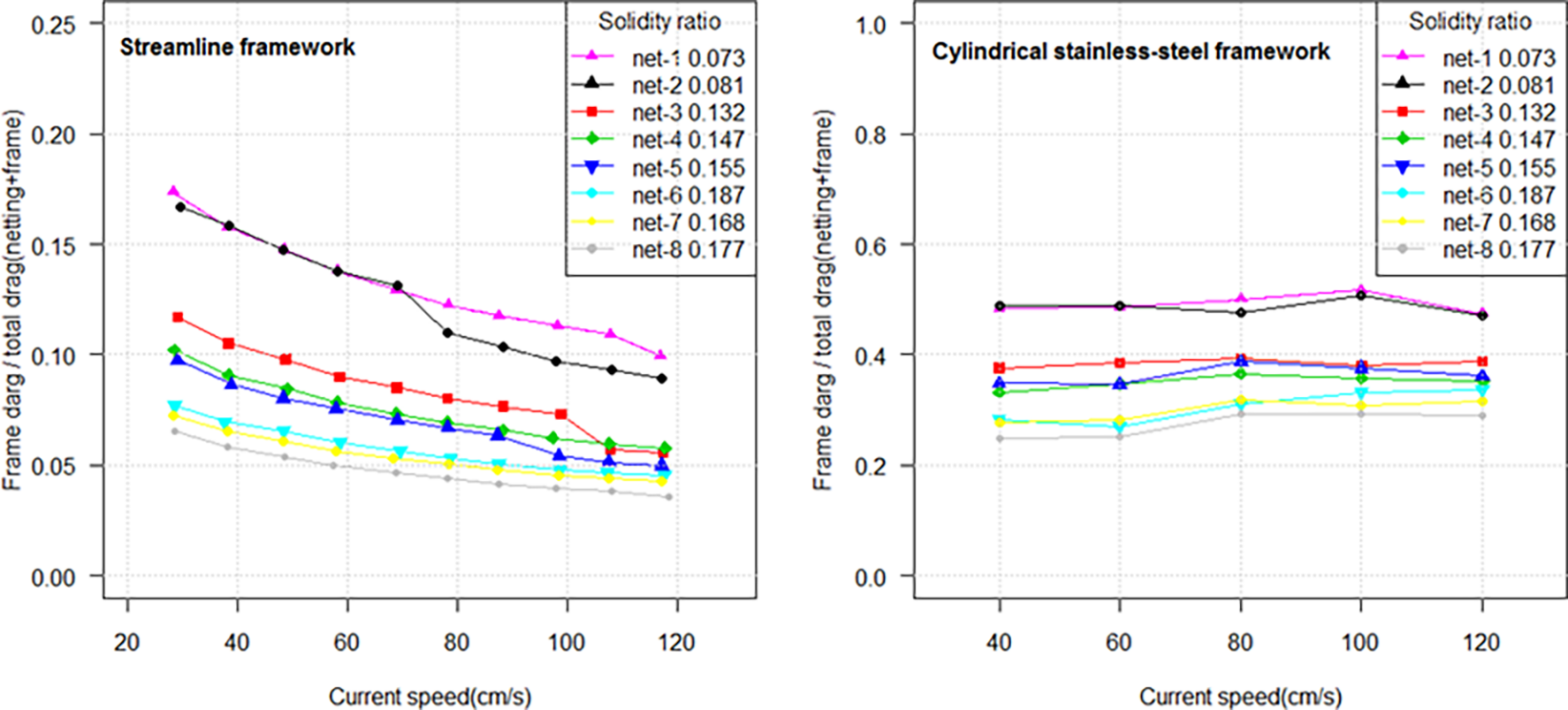
The ratio of frame drag to total drag under different flow velocities.

In order to provide reliable data, it should make sure that the experimental frames are fit for use and that the measuring process is correct. In the inclined netting panel experiment, we measured the drag of netting at attack angle of 90° (normal to the flow) using a cylindrical stainless-steel frame. We compared the ratio of frame drag to total drag (netting drag + frame drag) between cylindrical stainless-steel frame and streamlined frame under different flow velocities (Fig 15). The frame drag-total drag ratios cover from 0.03 to 0.17 when using a streamlined frame, and the ratio exhibited lower value at higher flow velocity and lager solidity ratio. Applying a streamlined frame could prevent turbulent or vortices to experiment netting by flow velocity. However, the ratios of frame drag to total drag were greater than 0.2 when using cylindrical stainless steel frame, which is greater recommended 0.1 [1]. Therefore, we recommend the use of streamlined frames when studying hydrodynamic forces of netting panel normal or parallel to the incoming flow.

## Conclusions

Experiments were carried out to investigate the effects of solidity ratio, flow velocity and knot type on the hydrodynamic characteristics of plane netting in uniform flow at different attack angles. The following conclusions can be drawn from the experiments and subsequent analysis:

1. Both Reynolds number and solidity ratio have a significant effect on hydrodynamic coefficients, and solidity ratio is negatively correlated with the parallel drag coefficient, but the opposite is true when the netting panel is set normal to the flow.
2. The lift coefficient reached the culminating point at an attack angle of 50°, and the dual effect of solidity ratio on inclined drag coefficient was observed.
3. The drag coefficient of knotted netting when setting normal to the flow is 1.23~1.35 times greater than that of knotless netting, so the knot type should be taken into account in hydrodynamic model development.
4. The drag coefficients of knotless PE netting are dominant at low Reynolds number (Re<2200), but the drag coefficients of PA netting are greater than that of PE netting at Reynolds number higher than 2200.
5. Compared to several previously published hydrodynamic coefficient models, there appeared to be some differences in the hydrodynamic coefficient–this may have been attributable to differences in the hypothetical mathematical model, experimental netting (netting materials, knot type, mesh angle, woven pattern and materials roughness), and experiment setup. Further studies are necessary to develop a comprehensive hydrodynamic models for fabric nets.

## Supporting information

S1 File The original hydrodynamic data of netting panels with various solidity ratios under different attack angles and current speeds. A: netting is set inclined to flow, B: netting is set normal to flow, C: netting is set parallel to flow.
(RAR)
